# Distinct microbiome composition and reduced interactions in patients with pancreatic cancer

**DOI:** 10.3389/fmicb.2025.1555479

**Published:** 2025-06-20

**Authors:** Bomi Kim, Sujin Oh, Soomin Yang, Jinwoo Ahn, Kwangrok Jung, Jong-Chan Lee, Jin-Hyeok Hwang, Cheol Min Shin, Hyo-Jung Lee, Hye Seung Lee, Jaihwan Kim, Kyoung Un Park

**Affiliations:** ^1^Department of Internal Medicine, Seoul National University Bundang Hospital, Seoul National University College of Medicine, Seongnam, Republic of Korea; ^2^Department of Laboratory Medicine, Seoul National University College of Medicine, Seoul, Republic of Korea; ^3^Department of Periodontology, Section of Dentistry, Seoul National University Bundang Hospital, Seoul National University School of Dentistry, Seongnam, Republic of Korea; ^4^Department of Pathology, Seoul National University Hospital, Seoul National University College of Medicine, Seoul, Republic of Korea; ^5^Department of Laboratory Medicine, Seoul National University Bundang Hospital, Seongnam, Republic of Korea

**Keywords:** pancreatic cancer, microbiome, saliva, feces, blood

## Abstract

**Introduction:**

The results of microbiome composition in patients with malignancy have been inconsistent across studies and are affected by various factors. This study aimed to identify microbiome composition of saliva, feces, and blood in patients with pancreatic cancer.

**Results:**

Overall, 31 patients with pancreatic cancer and 24 healthy controls were sex- and age-matched. Microbiome analysis of saliva, fecal, and blood samples was conducted using 16S rRNA amplicon sequencing. Baseline characteristics were comparable between patients and controls. Saliva showed insignificant difference in alpha diversity (*p* = 0.42), whereas feces and blood exhibited a significant difference in Shannon’s index (feces: 6.19 vs. 6.52, *p* = 0.013; blood: 8.00 vs. 7.49, *p* < 0.001) between patients and controls. Beta diversity analysis revealed significant differences between saliva, fecal, and blood samples (*p* = 0.014, 0.001, and 0.001, respectively). Distinct microbiome compositions were identified in patients, with higher abundance of *Lactobacillus*, *Enterobacter*, and *Prevotella* in saliva, fecal, and blood samples, respectively. Based on microbial network analysis, patients with pancreatic cancer showed lower clustering coefficient (71% vs. 99%) and higher average path length (1.67 vs. 0.68) than healthy controls, suggesting a more compact network and stronger microbial interactions in healthy controls.

**Conclusion:**

This study identified a distinctive microbiome in patients with pancreatic cancer, indicating the presence of *Lactobacillus*, *Enterobacter*, and *Prevotella*. A less condensed and robust microbial interaction network was observed in blood samples of patients with pancreatic cancer. These findings provide a basis for research on the connection between the microbiome and pancreatic cancer.

## 1 Introduction

The incidence of pancreatic cancer is increasing, and it is expected to become the second leading cause of cancer-related deaths worldwide by 2030 (Rahib et al., 2014; Siegel et al., 2023). The average 5-year survival rate in pancreatic cancer has reached approximately 12%, and the 5-year survival rate in patients with localized disease is only 44% (Siegel et al., 2023).

The tumor microenvironment (TME) plays an important role in tumor growth, metastasis, and disease characteristics. The TME comprises various cell types that interact with each other (Hanahan and Weinberg, 2011). The microbiome, a constituent of the TME, modulates the inflammatory response, which can drive carcinogenesis (Zambirinis et al., 2014; Yu et al., 2021). Microbiome analysis has been reported to be a potential diagnostic tool for malignancies (Poore et al., 2020).

Studies on the microbiome of saliva and feces from patients with pancreatic cancer have reported variations in diversity and taxonomy (Torres et al., 2015; Ren et al., 2017; Half et al., 2019; Zhou et al., 2021; Nagata et al., 2022; Petrick et al., 2022). This inconsistency is attributable to differences between individuals, sample sites, and lifestyle variables, such as diet, medication, and familial factors (Song et al., 2013; Gilbert et al., 2018).

Although human blood is considered sterile, modern sequencing techniques have detected bacterial genetic material even in the blood of healthy individuals (Potgieter et al., 2015). Distinct blood microbial profiles have been reported in several malignancies (Cho et al., 2019; Poore et al., 2020; An et al., 2022; Woerner et al., 2022; Cheng et al., 2023). This underscores the importance of blood microbiome analysis in cancer. However, studies examining the blood microbiome in pancreatic cancer remain limited and underexplored.

There are limited studies on microbiome composition in patients with pancreatic cancer. In particular, comprehensive analyses remain scarce, especially those that include blood samples. This study aimed to characterize microbiome composition of saliva, fecal, and blood samples in patients with pancreatic cancer and to explore microbial interaction networks beyond compositional differences.

## 2 Materials and methods

### 2.1 Patients

We collected saliva, fecal, and blood samples from patients with pancreatic cancer in a single tertiary teaching hospital between December 2019 and May 2022. Samples from healthy controls were acquired from the Periodontal Human Specimen Storage Registry at Seoul National University Bundang Hospital, with approval for secondary research. We reviewed a database of patients with pancreatic cancer. A survey was conducted among patients with pancreatic cancer and healthy controls to obtain information on the underlying medical conditions and oral care practices. When assessing smoking history, individuals who had quit for ≥6 months were categorized as the nonsmoking group and those who had quit within the past 6 months were classified as the smoking group (Boutou et al., 2008; Walton et al., 2020). Experienced periodontists evaluated periodontal health parameters, including periodontal probing depth and missing teeth count.

Written informed consent was obtained from all participants prior to inclusion in the study. This study was conducted in accordance with the Declaration of Helsinki and was approved by the Institutional Review Board of the Seoul National University Bundang Hospital (no. B-2110-714-303).

### 2.2 Sample collection and preparation

Patients with pancreatic cancer and healthy controls were instructed to abstain from oral hygiene practices for a minimum of 2 h before saliva collection. Fecal samples were self-collected by the participants using a sterile spatula, placed in a sterile container designed for feces, and immediately stored in a freezer until transportation on ice to the laboratory. Venous blood samples were aseptically collected by trained personnel. Upon arrival at the laboratory, all samples, excluding fecal samples, were stored at −80°C until DNA extraction. DNA was extracted from 1 mL of thawed sample using QIAamp DNA Microbiome Kit (QIAGEN, Venlo, the Netherlands), following the manufacturer’s protocol.

### 2.3 16S rRNA amplicon sequencing

DNA quality was assessed using Qubit dsDNA HS Assay Kits (Thermo Fisher Scientific Inc., Waltham, MA, United States). Polymerase chain reaction (PCR) targeting V3 and V4 hypervariable regions of 16S rRNA genes was conducted using KAPA HiFi HotStart ReadyMix PCR Kit (Roche, Basel, Switzerland) following the manufacturer’s instructions. The primer sequences used for PCR amplification were as follows: 519F: 5′-CCTACGGGNGGCWGCAG-3′ and 806R: 5′-GACTACHVGGGTATCTAATCC-3′. Libraries were constructed utilizing Nextera XT DNA Library Preparation Kit (Illumina Inc., San Diego, CA, United States), and the amplified samples were pooled to achieve a final loading concentration of 8 pM. Subsequently, paired-end (2 × 300 bp) sequencing was performed using the MiSeq platform (Illumina).

### 2.4 Data analysis and visualization

The reads were processed using a Divisive Amplicon Denoising Algorithm (DADA2)-based pipeline within the Quantitative Insights Into Microbial Ecology (QIIME2) 22.2 platform. This process involved generation of an amplicon sequence variant (ASV) table through quality-based filtering and trimming, read deduplication, ASV inference, paired-end merging, and chimera removal. ASVs were taxonomically classified against the 99% SILVA rRNA taxonomy. To rectify artifactual biases, feature tables were normalized via rarefaction.

For alpha diversity analysis, including observed features, Shannon’s entropy, Pielou’s evenness, and Faith’s phylogenetic diversity were calculated. To evaluate dissimilarities between microbial compositions of each sample, beta diversity indices, such as the Bray–Curtis index, and unweighted UniFrac distance were calculated. Principal coordinate analysis (PCoA) was used to visualize overall trends in sample dissimilarities. PERMANOVA based on Bray–Curtis dissimilarity was performed with BMI included as a covariate to assess group differences in microbial composition after adjustment. Moreover, permutation multivariate analysis of variance was performed to quantify the strength of associations between microbial composition and sample variables. To identify differentially abundant taxa between sample groups, we performed analysis of compositions of microbiomes with bias correction (ANCOM-BC), which can estimate unknown sampling fractions and correct for bias resulting from differences through a log-linear regression model. Then, we used Phylogenetic Investigation of Communities by Reconstruction of Unobserved States 2 (PICRUSt2), which can predict microbial functions based on 16S marker gene sequences. To examine variations in microbial metabolism, predicted orthologs were collapsed into the Kyoto Encyclopedia of Genes and Genomes pathways, followed by differential abundance (DA) analysis using ANOVA-Like Differential Expression tool version 2 (ALDEx2). Correction for multiple testing was performed using the Benjamini–Hochberg method; thus, *Q*-values of <0.05 were considered to indicate statistical significance for both DA methods.

To examine interactions between microbiomes, co-occurrence network analysis was performed using sparse inverse covariance estimation for ecological association inference (SPIEC-EASI) via graphical lasso algorithms. Signed distance was computed to transform associations into dissimilarities. Topological properties of networks, including clustering coefficient, average path length, average dissimilarity, modularity, edge density, and positive edge ratio, were examined using the *igraph* package in R. Only nodes with >3 degrees are shown in the figures.

Statistical analyses and data visualization were performed using R software (ver. 4.1.2; R Development Core Team, Vienna, Austria). QIIME artifacts were imported into the R environment using the *qiime2R* package and then transformed into phyloseq objects using the *phyloseq* package. Centered log-ratio transformation of raw feature counts was performed before conducting statistical analyses of microbial abundance. The Wilcoxon rank-sum test was conducted to compare nonparametric distributions of alpha diversity between sample groups, and *p-*values of <0.05 were considered to indicate statistical significance.

## 3 Results

### 3.1 Baseline characteristics

Study participants included 31 patients with pancreatic cancer and 24 healthy controls. No significant difference was noted in the baseline characteristics between the two groups (Table 1). The median ages of patients with pancreatic cancer and healthy controls were 69 and 65 years, respectively (*p* = 0.81). In total, males constituted 58.1% (*n* = 18) and 66.7% (*n* = 16) of patients with pancreatic cancer and healthy controls, respectively (*p* = 0.61). The prevalence rates of diabetes mellitus (*p* = 0.31), smoking history (*p* = 0.93), drinking history (*p* = 0.20), and history of antibiotics use (*p* = 0.06) were not significantly different between the two groups. Although body mass index was significantly different between the two groups, none of the groups met the criteria for obesity (median body mass index: 21.7 and 24.7 kg/m^2^ for patients with pancreatic cancer and healthy controls, respectively). Pancreatic cancer was more frequent in the head than in the body or tail (*n* = 17, 54.8% vs. *n* = 14, 45.2%). In total, 14 (45.2%) tumors were resectable, 7 (22.6%) were borderline resectable, 5 (16.1%) were locally advanced, and 5 (16.1%) were metastatic. The median serum level of carbohydrate antigen 19–9 (CA 19–9) was 41.0 U/mL. Periodontal information revealed no difference in daily toothbrushing frequency (≤2 times a day: 51.9% vs. 40.0%, *p* = 0.52), median severity of severe periodontitis (4.0 vs. 4.0, *p* = 0.91), and number of missing teeth (1.5 vs. 2.0, *p* = 0.42) between the two groups.

**Table 1:** Baseline characteristics and periodontal information between the patient with pancreatic cancer and healthy control.

	Pancreatic cancer (*n* = 31, %)	Healthy control (*n* = 24, %)	*p*-value
Median age (year) (range)	69 (36–83)	65 (36–80)	0.81
Male	18 (60.0)	16 (66.7)	0.61
Diabetes (*n* = 31/23)	8 (25.8)	4 (17.4)	0.31
Smoking (*n* = 25/23)	2 (8.0)	2 (8.7)	0.93
Alcohol (*n* = 25/12)	9 (36.0)	7 (58.3)	0.20
BMI (SD) (kg/m^2^) (*n* = 31/17)	21.7 (2.2)	24.7(2.7)	<0.001
History of antibiotic use	5 (16.1)	0 (0.0)	0.06
Tumor location and resectability			
Head/Body/Tail	17 (54.8)/9 (29.0)/5 (16.1)		
Resectable/Borderline resectable/Locally advanced/metastatic	14 (45.2)/7 (22.6)/5 (16.1)/5 (16.1)		
Median CA 19–9 (SD) (U/ml)	41.0 (6,131.4)		
Periodontal information			
Tooth brushing/day ≤ 2 (*n* = 27/10)	14 (51.9)	4 (40.0)	0.52
Median severe periodontitis (SD)	4.0 (10.8)	4.0 (10.7)	0.91
Severe periodontitis	22 (73.3)	17 (70.8)	1.00
Median missing (SD)	1.5 (7.4)	2.0 (4.1)	0.42
Missing	20 (66.7)	16 (66.7)	1.00
Median missing & severe periodontitis (SD)	7.5 (13.0)	5.5 (12.6)	0.33

BMI, body mass index; SD, Standard Deviation; CA 19–9, Carbohydrate antigen 19–9.

### 3.2 Relative abundance of microbiota at phylum and genus levels

**Figure 1:**
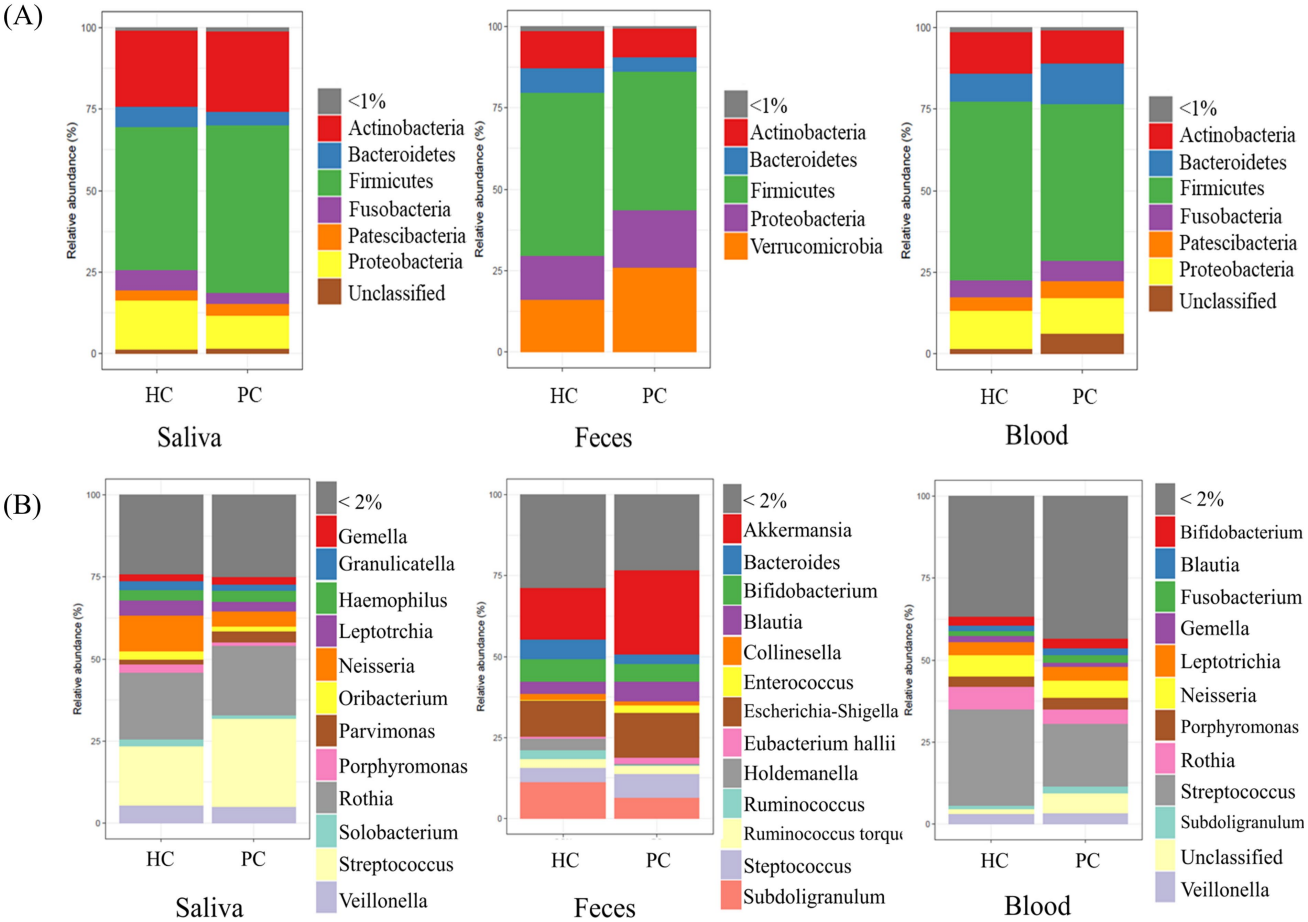
Relative abundance in each specimen. Bar plots represent the relative abundance of predominant microbiota constituents at the phylum **(A)** and genus **(B)** levels for each sample. HC, healthy control; PC, patient with pancreatic cancer.

The relative abundance of the predominant microbiota was analyzed at the phylum and genus levels in saliva, fecal, and blood samples from patients with pancreatic cancer and healthy controls (Figures 1A,B). At the phylum level, Firmicutes was dominant in the saliva, fecal, and blood samples of patients with pancreatic cancer and healthy controls. Saliva samples exhibited a high abundance of Actinobacteria, whereas fecal samples showed a prevalence of Verrucomicrobia. At the genus level, distinct variations in microbial composition were observed between saliva, fecal, and blood samples of the two groups. PCoA results of unweighted UniFrac distance confirmed differences in microbial composition of saliva, fecal, and blood samples between patients with pancreatic cancer and healthy controls (Supplementary Figure 1).

### 3.3 Alpha and beta diversities

**Figure 2:**
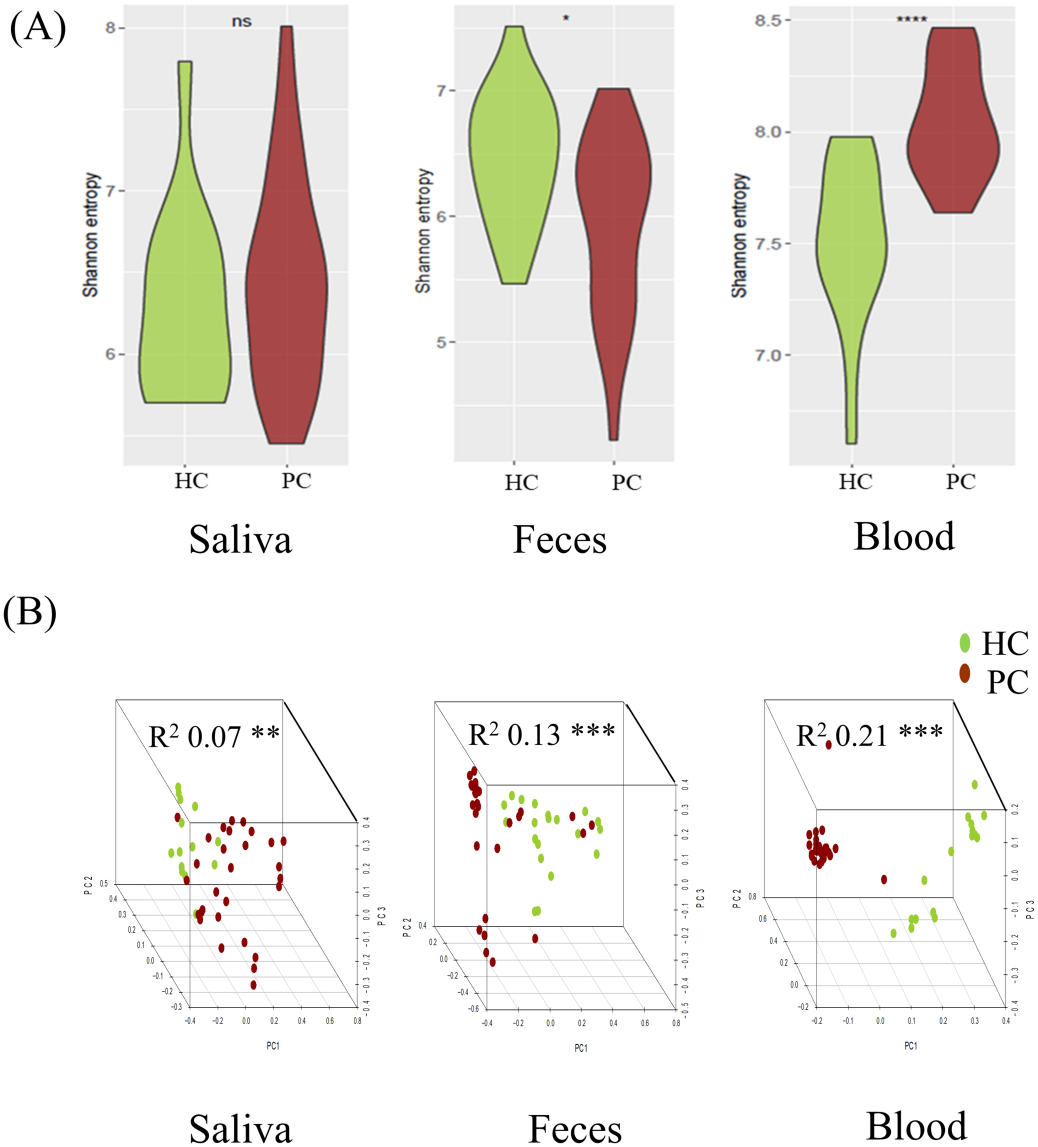
Comparative analysis of microbial diversity between patients with pancreatic cancer and matched healthy controls in each specimen. Alpha diversity, measured via Shannon’s entropy, was higher in fecal samples and lower in blood samples of healthy controls. **(A)** Beta diversity analysis using the Bray–Curtis distance indicated significant differences among sample types, allowing differentiation between patients with pancreatic cancer and healthy controls **(B)**. HC, healthy control; PC, patient with pancreatic cancer. Statistical significance (*p < 0.05, **p < 0.01, ***p < 0.001, ****p < 0.0001).

No significant difference was observed in the alpha diversity indices between saliva samples of patients with pancreatic cancer and those of healthy controls (median Shannon index: 6.45 vs. 6.22; *p* = 0.42). However, Shannon indices of fecal samples were higher in healthy controls than in patients with pancreatic cancer (median Shannon index: 6.19 vs. 6.52; *p* = 0.013). The blood microbiome showed higher richness and evenness in patients with pancreatic cancer than in healthy controls (median Shannon index: 8.00 vs. 7.49; *p* < 0.001; Figure 2A).

Beta diversity analysis using the Bray–Curtis distance revealed significant differences in microbial composition of saliva, fecal, and blood samples between patients with pancreatic cancer and healthy controls (saliva: *R*^2^ = 0.07, *p* = 0.014; feces: *R*^2^ = 0.13, *p* = 0.001; blood: *R*^2^ = 0.21, *p* = 0.001) (Figure 2B).

PERMANOVA based on Bray–Curtis dissimilarity showed no significant association between BMI and microbial composition in saliva (*R*^2^ = 0.025, *p* = 0.463), but significant associations in fecal (*R*^2^ = 0.038, *p* = 0.022) and blood samples (*R*^2^ = 0.063, *p* = 0.003). After adjusting for BMI, group differences between patients with pancreatic cancer and controls remained significant (fecal: *R*^2^ = 0.105, *p* = 0.001; blood: *R*^2^ = 0.165, *p* = 0.001).

### 3.4 Differentially abundant taxa between patients with pancreatic cancer and healthy controls

**Figure 3:**
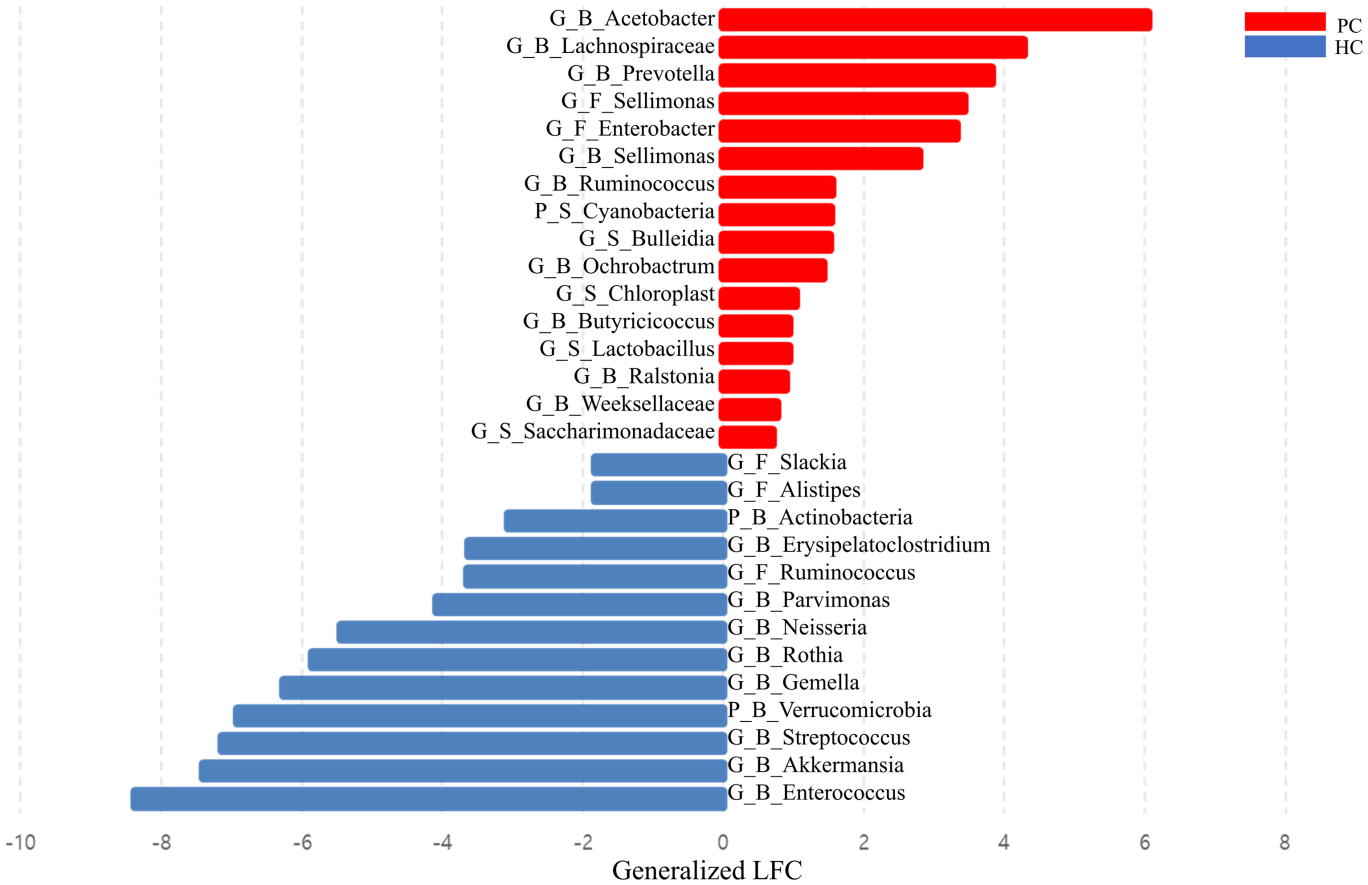
Bar plot showing the effect size of the difference between the abundance of each taxon in patients with pancreatic cancer and healthy controls. Effect sizes were estimated via differential abundance analysis using analysis of compositions of microbiomes with bias correction (ANCOM-BC) and expressed as log-fold change divided by the estimate of standard error. HC, healthy control; PC, patient with pancreatic cancer; P, phylum level; G, genus level; B, blood sample; F, fecal sample; LFC, log-fold change.

DA analysis revealed distinct microbial profiles between the two groups. Saliva samples of patients with pancreatic cancer had a significantly higher abundance of Cyanobacteria, *Bulleidia*, *Lactobacillus*, and *Saccharimonadaceae* than those of healthy controls. Furthermore, *Enterobacter* and *Sellimonas* were more abundant in the fecal samples of patients with pancreatic cancer than in those of healthy controls, whereas *Alistipes*, *Ruminococcus*, and *Slackia* were more abundant in the fecal samples of healthy controls than in those of patients with pancreatic cancer. *Acetobacter*, *Butyricicoccus*, *Ochrobactrum*, *Prevotella*, *Ralstonia*, *Ruminococcus*, *Sellimonas*, *Weeksellaceae*, and *Lachnospiraceae* were enriched in the blood samples of patients with pancreatic cancer, whereas Actinobacteria, Verrucomicrobia*, Akkermansia*, *Enterococcus*, *Erysipelatoclostridium*, *Gemella*, *Neisseria*, *Parvimonas*, *Rothia*, and *Streptococcus* were enriched in the blood samples of healthy controls (Figure 3).

### 3.5 Microbial interactions in blood samples of patients with pancreatic cancer

**Figure 4:**
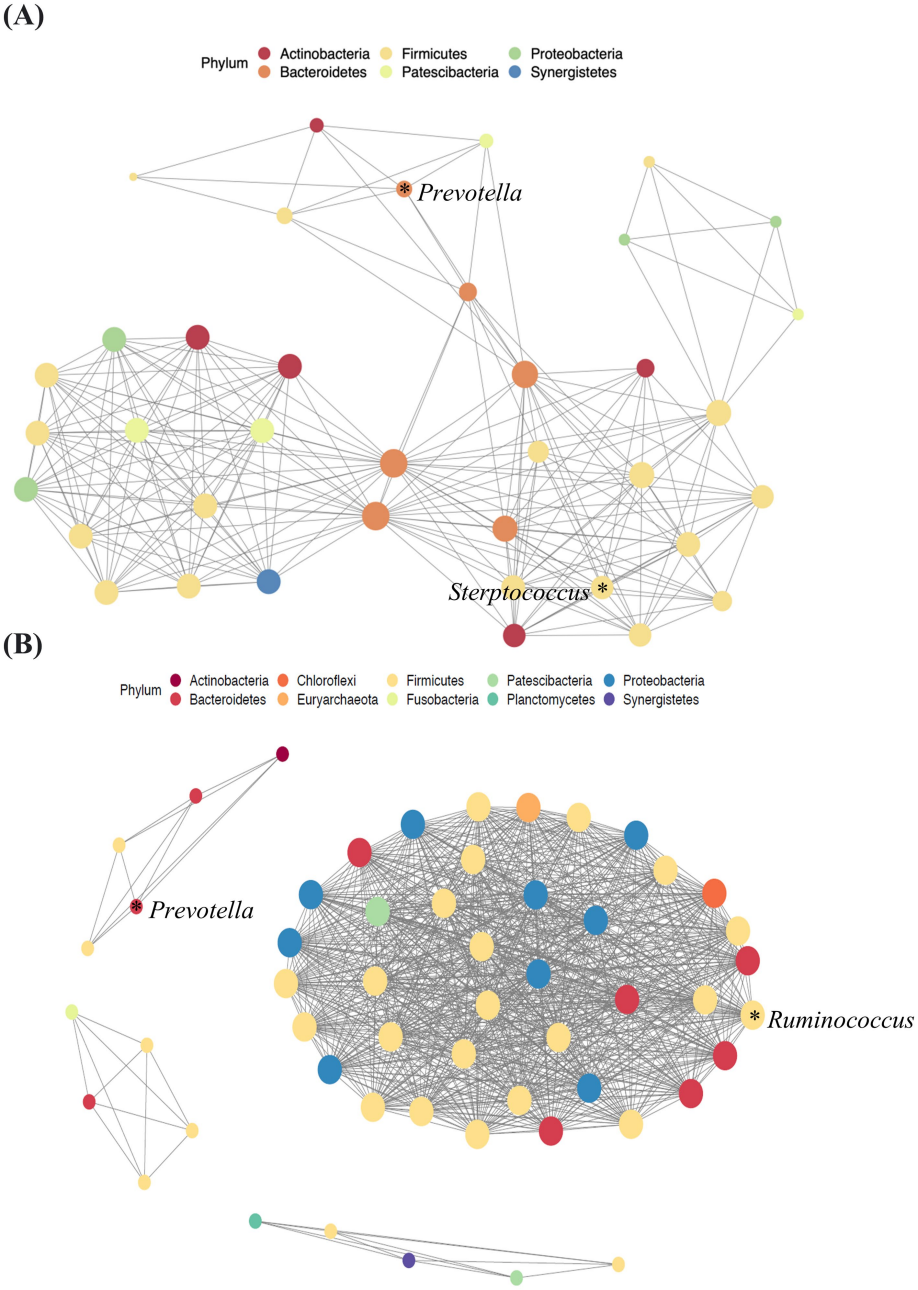
Network analysis of blood samples from patients with pancreatic cancer **(A)** and healthy controls **(B)**. Lines between dots indicate the significant correlation of species (*p* < 0.05). The size of the node is proportional to the relative abundance of species. The nodes are colored according to the phylum to which the species belongs.

The microbial interactions in blood samples differed between patients with pancreatic cancer and healthy controls (Table 2; Figure 4). The co-occurrence network of the blood microbiome had a higher clustering coefficient for healthy controls (99%) than for patients with pancreatic cancer (71%). The average path length, a metric indicating the compactness and strength of microbial interactions, was calculated by determining the average number of steps along the shortest paths for all possible pairs of network nodes. The average path length was lower in healthy controls (0.68) than in patients with pancreatic cancer (1.67). This suggests a more compact network and stronger microbial interactions in healthy controls than in patients with pancreatic cancer (Table 2).

**Table 2:** Comparison of network topological properties between patients with pancreatic cancer and healthy controls.

	Pancreatic cancer	Healthy control
Clustering coefficient	0.711	0.992
Modularity	0.487	0.150
Positive edge percentage	74.340	99.378
Edge density	0.017	0.053
Natural connectivity	0.010	0.049
Average dissimilarity	0.994	0.983
Average path length	1.670	0.684

This analysis revealed a notable prevalence of interactions, particularly that of Bacteroidetes with other microbiota, in the blood samples of patients with pancreatic cancer (Figure 4A). The microbial network of healthy controls revealed that Firmicutes was the most abundant key microorganism at the phylum level in blood samples (Figure 4B).

## 4 Discussion

Studies have analyzed the differences in the microbiome of patients with pancreatic cancer and healthy controls. However, the results have been inconsistent, and studies focusing on blood samples are limited. A comprehensive PubMed search identified eight microbiome studies on saliva samples (Torres et al., 2015; Olson et al., 2017; Fan et al., 2018; Lu et al., 2019; Vogtmann et al., 2020; Wei et al., 2020; Chen et al., 2023), seven on fecal samples (Ren et al., 2017; Half et al., 2019; Matsukawa et al., 2021; Kartal et al., 2022; Chen et al., 2023; Hashimoto et al., 2023; Yang et al., 2023), and none on blood samples from patients with pancreatic cancer (Table 3). By comparing the significant taxa identified in our study with those reported in other studies, we identified similarities. Saliva samples of patients with pancreatic cancer exhibited a significant increase in the abundance of *Lactobacillus*, consistent with the finding of other studies. *Enterobacter* was significantly abundant in the fecal samples of patients with pancreatic cancer in our study as well as other studies. The current study compared the microbiome profiles of patients with pancreatic cancer and healthy controls based on 16S rRNA sequencing of saliva, fecal, and blood samples. This study identified features that differentiated the microbial composition of patients with pancreatic cancer from that of healthy controls.

**Table 3:** Studies on the association between microbiome and pancreatic cancer at oral (A), stool (B), and blood (C).

Author (publication year, country)	Sample size	alpha-diversity	Result: increased	Result: decreased
(A)				
Wei et al. (2020, China)	PC 41/HC 69	↓	*Bacilli, Streptococcus, Firmicutes, Actinomyces, Rothia, Leptotrichia, Lactobacillus, Escherichia_coli, Enterobacteriales*	*Selenomonas, Porphyromnas, Prevotella, Capnocytophaga, Alloprevotella, Tannerella, Neisseria*
Fan et al. (2018, United States)	PC 361/HC 371		*Porphyromonas gingivalis, Aggregatibacter actinomycetemcomitans*	*Fusobacteria, Leptotrichia*
Lu et al. (2019, China)	PC 30/HC 25	↑	*Leptotrichia, Fusobacterium, Rothia, Actinomyces, Corynebacterium, Atopobium, Peptostreptococcus, Catonella, Oribacterium, Filifactor, Campylobacter, Moraxella, Tannerella*	*Haemophilus, Porphyromonas, Paraprevotella*
Olson et al. (2017, Canada)	PC 40/IPMN 39/HC 58	↓	*Firmicutes, Bacilli, Lactobacillales, Streptococcaceae, Streptococcus, Streptococcus thermophilus*	*Proteobacteria, Gammaproteobacteria, Pasteurellales, Pasteurellaceae, Haemophilus, Haemophilus parainfluenzae, Betaproteobacteria, Neisseriales, Neisseriaceae, Neisseria, Neisseria flavescens*
Chen et al. (2023, China)	PC 40/CP 15/HC 39		*Firmicutes, Verrucomicrobia, Veillonella, Peptostreptococcus, Akkermansia, Parvimonas, Solobacterium, Olsenella, Escherichia, Shigella*	
Vogtmann et al. (2020, Iran)	PC 273/HC 285	↑	*Enterobacteriaceae, Lachnospiraceae G7, Bacteroidaceae, Staphylococcaceae*	*Haemophilus*
Torres et al. (2015, United States)	PC 8/other diseases 78/HC 22		*Leptotrichia*, *Porphyromonas*	*Neisseria, Aggregatibacter*
Our study (2024, Korea)	PC 31/HC 24		*Cyanobacteria*, *Bulleidia, Lactobacillus, Saccharimonadaceae, Chloroplast*	
(B)				
Matsukawa et al. (2021, Japan)	PC 24/HC 18		*Klebsiella pneumoniae, Clostridium bolteae, C. symbiosum, Streptococcus mutans, Alistipes shahii, Bacteroides species, Parabacteroides species, Lactobacillus*	
Ren et al. (2017, China)	PC 24/HC 18		*Prevotella, Veillonella, Klebsiella, Selenomonas, Hallella, Enterobacter, Cronobacter*	*Gemmiger, Bifidobacterium, Coprococcus, Clostridium IV, Blautia, Flavonifractor, Anaerostipes, Butyricicoccus, Dorea*
Yang et al. (2023, China)	PC 44/HC 50	↑	*Streptococcus*	
Chen et al. (2023, China)	PC 40/CP 15/HC 39		*Prevotella, Coprobacter, Proteobacteria, Peptostreptococcus, Actinomyces, Bifidobacterium, Campylobacter, Coprobacillus, Escherichia-Shigella*	
Hashimoto et al. (2023, Japan)	PC 5/HC 68		*Actinomyces, Streptococcus, Veillonella, Lactobacillus*	*Anaerostipes*
Kartal E et al. (2022, EU)	PC 57/CP 29/HC 50		*Veillonella atypica, Fusobacterium nucleatum/hwasookii, Alloscardovia omnicolens*	*Romboutsia timonensis, Faecalibacterium prausnitzii, Bacteroides coprocola, Bifidobacterium bifidum*
Half et al. (2019, Israel)	PC 30/pre-cancerous lesions 6/HC 13/NAFLD 16		*Bacteroidetes*, *Veillonellaceae*, *Akkermansia, Odoribacter*	*Firmicutes, Clostridiacea, Lachnospiraceae, Ruminococcaceae*
Our study (2024, Korea)	PC 31/HC 24	↓	*Enterobacter, Sellimonas*	*Alistipes, Ruminococcus, Slackia*
(C)				
Our study (2024, Korea)	PC 31/HC 24	↑	*Acetobacter, Butyricicoccus, Ochrobactrum, Prevotella, Ralstonia, Ruminococcus, Sellimonas, Weeksellaceae Lachnospiraceae*	*Actinobacteria, Verrucomicrobia, Akkermansia, Enterococcus, Erysipelatoclostridium, Gemella, Neisseria, Parvimonas, Rothia, Streptococcus*

HC, healthy controls; PC, patients with pancreatic cancer; IPMN, intraductal papillary mucinous neoplasm; CP, Chronic pancreatitis; NAFLD, non-alcoholic fatty liver disease.

Previous studies on saliva samples have shown inconsistent findings regarding alpha diversity in patients with pancreatic cancer (Olson et al., 2017; Lu et al., 2019; Vogtmann et al., 2020; Wei et al., 2020). However, alpha diversity showed no significant difference between patients with pancreatic cancer and healthy controls in the current study, consistent with the findings of a few studies (Torres et al., 2015; Chen et al., 2023). Fecal samples of patients with pancreatic cancer exhibited decreased alpha diversity and a significant difference in beta diversity, consistent with the findings of other studies (Li J. J. et al., 2021; An et al., 2022; Kohi et al., 2022; Sidiropoulos et al., 2024).

While it is well-known that BMI, especially overweight, can influence the composition of the microbiome (Xu et al., 2022), it is also established that pancreatic cancer patients often have significantly poorer BMI (Bachmann et al., 2009). Notably even after adjusting for BMI using PERMANOVA, the differences in microbial composition between groups remained significant, supporting the interpretation that disease-related factors play a larger role in shaping the microbiome than BMI alone.

Network analysis of the blood microbiome revealed a higher clustering coefficient and lower average path length in healthy controls than in patients with pancreatic cancer, indicating greater complexity and strength of microbial interactions. Microbial ecosystems with higher clustering coefficients have been shown to exhibit greater stability and metabolic activity, supporting the notion that the observed reduction in our cancer cohort may reflect ecological fragility of the microbiome (Guo et al., 2022). Therefore, in patients with pancreatic cancer, the complexity and compactness of microbial interactions are reduced. This result is consistent with that of other studies reporting similar patterns in microbial interaction network in other cancer types (Liu et al., 2019; Zhou et al., 2020; Uriarte-Navarrete et al., 2021).

This study found distinctive microbiomes, such as *Lactobacillus, Enterobacter*, and *Prevotella* in saliva, fecal, and blood samples of patients with pancreatic cancer, respectively. *Lactobacillus* was consistently elevated in the saliva of patients with pancreatic cancer, which is consistent with the findings of other studies (Wei et al., 2020). In contrast, an increased abundance of *Lactobacillus* was reported in the fecal samples of patients with pancreatic cancer (Kartal et al., 2022). In a mouse model of pancreatic cancer, *Lactobacillus* influenced macrophage activity, potentially contributing to rapid disease progression and mortality (Hezaveh et al., 2022). In a comparison of saliva samples between patients with precancerous lesions and squamous cell carcinoma, *Lactobacillus* was more abundant in patients with cancer (Li Z. et al., 2021). The abundance of *Enterobacter* in the fecal samples of patients with pancreatic cancer was consistent with that reported in other studies (Ren et al., 2017; Nejman et al., 2020). *Enterobacter* was more abundant in tumor (Nejman et al., 2020; Kohi et al., 2022) and bile samples of patients with pancreatic cancer (Nejman et al., 2020). In a mouse study, *Enterobacter* induced chronic pancreatitis, elevating the risk of pancreatic cancer development (Maekawa et al., 2018). Consistently, Enterobacteriaceae was abundant in pancreatic cancer (Geller et al., 2017). *Prevotella* was more abundant in the saliva samples of healthy controls than in those of patients with pancreatic cancer (Wei et al., 2020). In contrast, *Prevotella* had a higher prevalence in the tumors (Nejman et al., 2020) and feces (Ren et al., 2017) of patients with pancreatic cancer. These findings underscore the complex interplay between *Lactobacillus*, *Enterobacter*, and *Prevotella*, and cancer, warranting further investigation.

This study had several limitations. First, the study population was small, although it was comparable to other studies. Second, as this is a single center study, studies from several institutions are needed for generalizing the findings. Third, because this study conducted a cross-sectional microbiome analysis, additional experimental models must establish causality between microbial taxa and pancreatic cancer.

In conclusion, this study identified significant microbial taxa such as *Lactobacillus*, *Enterobacter*, and *Prevotella* in patients with pancreatic cancer. Network analysis revealed reduced complexity, strength, and compactness of microbial interaction patterns in the blood samples of patients with pancreatic cancer. Our findings can serve as a guide for future research on the complex connection between the microbiome and pancreatic cancer.

## Data Availability

Sequence data supporting the findings of this study have been deposited in the Sequence Read Archive (SRA) with the BioProject accession number: PRJNA1162657 (https://www.ncbi.nlm.nih.gov/bioproject/1162657).

## Supplementary material

SUPPLEMENTARY FIGURE 1 The microbial composition relationships between the sample types were assessed between healthy controls and patients with pancreatic cancer. Principal coordinate analysis (PCoA) was employed to visualize the relationships between samples using unweighted UniFrac distance matrices. HC, healthy control; PC, patient with pancreatic cancer. Differences in microbial composition between patients with pancreatic cancer and healthy controls.

## References

[ref1] An J. Kwon H. Lim W. Moon B. I. (2022). *Staphylococcus aureus*-derived extracellular vesicles enhance the efficacy of endocrine therapy in breast Cancer cells. J. Clin. Med. 11:2030. doi: 10.3390/jcm11072030, PMID: 35407638 PMC9000115

[ref2] Bachmann J. Ketterer K. Marsch C. Fechtner K. Krakowski-Roosen H. Buchler M. W.. (2009). Pancreatic cancer related cachexia: influence on metabolism and correlation to weight loss and pulmonary function. BMC Cancer 9:255. doi: 10.1186/1471-2407-9-255, PMID: 19635171 PMC2741486

[ref3] Boutou A. K. Tsiata E. A. Pataka A. Kontou P. K. Pitsiou G. G. Argyropoulou P. (2008). Smoking cessation in clinical practice: predictors of six-month continuous abstinence in a sample of Greek smokers. Prim. Care Respir. J. 17, 32–38. doi: 10.3132/pcrj.2008.00009, PMID: 18264649 PMC6619875

[ref4] Chen T. Li X. Li G. Liu Y. Huang X. Ma W.. (2023). Alterations of commensal microbiota are associated with pancreatic cancer. Int. J. Biol. Markers 38, 89–98. doi: 10.1177/03936155231166721, PMID: 37017014

[ref5] Cheng H. S. Tan S. P. Wong D. M. K. Koo W. L. Y. Wong S. H. Tan N. S. (2023). The blood microbiome and health: current evidence, controversies, and challenges. Int. J. Mol. Sci. 24:5633. doi: 10.3390/ijms24065633, PMID: 36982702 PMC10059777

[ref6] Cho E. J. Leem S. Kim S. A. Yang J. Lee Y. B. Kim S. S.. (2019). Circulating microbiota-based metagenomic signature for detection of hepatocellular carcinoma. Sci. Rep. 9:7536. doi: 10.1038/s41598-019-44012-w, PMID: 31101866 PMC6525191

[ref7] Fan X. Alekseyenko A. V. Wu J. Peters B. A. Jacobs E. J. Gapstur S. M.. (2018). Human oral microbiome and prospective risk for pancreatic cancer: a population-based nested case-control study. Gut 67, 120–127. doi: 10.1136/gutjnl-2016-312580, PMID: 27742762 PMC5607064

[ref8] Geller L. T. Barzily-Rokni M. Danino T. Jonas O. H. Shental N. Nejman D.. (2017). Potential role of intratumor bacteria in mediating tumor resistance to the chemotherapeutic drug gemcitabine. Science 357, 1156–1160. doi: 10.1126/science.aah5043, PMID: 28912244 PMC5727343

[ref9] Gilbert J. A. Blaser M. J. Caporaso J. G. Jansson J. K. Lynch S. V. Knight R. (2018). Current understanding of the human microbiome. Nat. Med. 24, 392–400. doi: 10.1038/nm.4517, PMID: 29634682 PMC7043356

[ref10] Guo B. Zhang L. Sun H. Gao M. Yu N. Zhang Q.. (2022). Microbial co-occurrence network topological properties link with reactor parameters and reveal importance of low-abundance genera. NPJ Biof. Microb. 8:3. doi: 10.1038/s41522-021-00263-y, PMID: 35039527 PMC8764041

[ref11] Half E. Keren N. Reshef L. Dorfman T. Lachter I. Kluger Y.. (2019). Fecal microbiome signatures of pancreatic cancer patients. Sci. Rep. 9:16801. doi: 10.1038/s41598-019-53041-4, PMID: 31727922 PMC6856127

[ref12] Hanahan D. Weinberg R. A. (2011). Hallmarks of cancer: the next generation. Cell 144, 646–674. doi: 10.1016/j.cell.2011.02.013, PMID: 21376230

[ref13] Hashimoto S. Tochio T. Funasaka K. Funahashi K. Hartanto T. Togashi Y.. (2023). Changes in intestinal bacteria and imbalances of metabolites induced in the intestines of pancreatic ductal adenocarcinoma patients in a Japanese population: a preliminary result. Scand. J. Gastroenterol. 58, 193–198. doi: 10.1080/00365521.2022.2114812, PMID: 36036243

[ref14] Hezaveh K. Shinde R. S. Klotgen A. Halaby M. J. Lamorte S. Ciudad M. T.. (2022). Tryptophan-derived microbial metabolites activate the aryl hydrocarbon receptor in tumor-associated macrophages to suppress anti-tumor immunity. Immunity 55:e328, 324–340. doi: 10.1016/j.immuni.2022.01.006 PMC8888129 35139353

[ref15] Kartal E. Schmidt T. S. B. Molina-Montes E. Rodriguez-Perales S. Wirbel J. Maistrenko O. M.. (2022). A faecal microbiota signature with high specificity for pancreatic cancer. Gut 71, 1359–1372. doi: 10.1136/gutjnl-2021-324755, PMID: 35260444 PMC9185815

[ref16] Kohi S. Macgregor-Das A. Dbouk M. Yoshida T. Chuidian M. Abe T.. (2022). Alterations in the duodenal fluid microbiome of patients with pancreatic Cancer. Clin. Gastroenterol. Hepatol. 20, e196–e227. doi: 10.1016/j.cgh.2020.11.006, PMID: 33161160 PMC8120597

[ref17] Li Z. Dou L. Zhang Y. He S. Zhao D. Hao C.. (2021). Characterization of the Oral and esophageal microbiota in esophageal precancerous lesions and squamous cell carcinoma. Front. Cell. Infect. Microbiol. 11:714162. doi: 10.3389/fcimb.2021.714162, PMID: 34604107 PMC8479167

[ref18] Li J. J. Zhu M. Kashyap P. C. Chia N. Tran N. H. Mcwilliams R. R.. (2021). The role of microbiome in pancreatic cancer. Cancer Metastasis Rev. 40, 777–789. doi: 10.1007/s10555-021-09982-2, PMID: 34455517 PMC8402962

[ref19] Liu X. Shao L. Liu X. Ji F. Mei Y. Cheng Y.. (2019). Alterations of gastric mucosal microbiota across different stomach microhabitats in a cohort of 276 patients with gastric cancer. EBioMedicine 40, 336–348. doi: 10.1016/j.ebiom.2018.12.034, PMID: 30584008 PMC6412016

[ref20] Lu H. Ren Z. Li A. Li J. Xu S. Zhang H.. (2019). Tongue coating microbiome data distinguish patients with pancreatic head cancer from healthy controls. J. Oral Microbiol. 11:1563409. doi: 10.1080/20002297.2018.1563409, PMID: 30728915 PMC6352935

[ref21] Maekawa T. Fukaya R. Takamatsu S. Itoyama S. Fukuoka T. Yamada M.. (2018). Possible involvement of Enterococcus infection in the pathogenesis of chronic pancreatitis and cancer. Biochem. Biophys. Res. Commun. 506, 962–969. doi: 10.1016/j.bbrc.2018.10.169, PMID: 30401562

[ref22] Matsukawa H. Iida N. Kitamura K. Terashima T. Seishima J. Makino I.. (2021). Dysbiotic gut microbiota in pancreatic cancer patients form correlation networks with the oral microbiota and prognostic factors. Am. J. Cancer Res. 11, 3163–3175. 34249452 PMC8263681

[ref23] Nagata N. Nishijima S. Kojima Y. Hisada Y. Imbe K. Miyoshi-Akiyama T.. (2022). Metagenomic identification of microbial signatures predicting pancreatic Cancer from a multinational study. Gastroenterology 163, 222–238. doi: 10.1053/j.gastro.2022.03.054, PMID: 35398347

[ref24] Nejman D. Livyatan I. Fuks G. Gavert N. Zwang Y. Geller L. T.. (2020). The human tumor microbiome is composed of tumor type-specific intracellular bacteria. Science 368, 973–980. doi: 10.1126/science.aay9189, PMID: 32467386 PMC7757858

[ref25] Olson S. H. Satagopan J. Xu Y. Ling L. Leong S. Orlow I.. (2017). The oral microbiota in patients with pancreatic cancer, patients with IPMNs, and controls: a pilot study. Cancer Causes Control 28, 959–969. doi: 10.1007/s10552-017-0933-8 28762074 PMC5709151

[ref26] Petrick J. L. Wilkinson J. E. Michaud D. S. Cai Q. Gerlovin H. Signorello L. B.. (2022). The oral microbiome in relation to pancreatic cancer risk in African Americans. Br. J. Cancer 126, 287–296. doi: 10.1038/s41416-021-01578-5, PMID: 34718358 PMC8770575

[ref27] Poore G. D. Kopylova E. Zhu Q. Carpenter C. Fraraccio S. Wandro S.. (2020). Microbiome analyses of blood and tissues suggest cancer diagnostic approach. Nature 579, 567–574. doi: 10.1038/s41586-020-2095-1, PMID: 32214244 PMC7500457

[ref28] Potgieter M. Bester J. Kell D. B. Pretorius E. (2015). The dormant blood microbiome in chronic, inflammatory diseases. FEMS Microbiol. Rev. 39, 567–591. doi: 10.1093/femsre/fuv013, PMID: 25940667 PMC4487407

[ref29] Rahib L. Smith B. D. Aizenberg R. Rosenzweig A. B. Fleshman J. M. Matrisian L. M. (2014). Projecting cancer incidence and deaths to 2030: the unexpected burden of thyroid, liver, and pancreas cancers in the United States. Cancer Res. 74, 2913–2921. doi: 10.1158/0008-5472.CAN-14-0155, PMID: 24840647

[ref30] Ren Z. Jiang J. Xie H. Li A. Lu H. Xu S.. (2017). Gut microbial profile analysis by MiSeq sequencing of pancreatic carcinoma patients in China. Oncotarget 8, 95176–95191. doi: 10.18632/oncotarget.18820, PMID: 29221120 PMC5707014

[ref31] Sidiropoulos T. Dovrolis N. Katifelis H. Michalopoulos N. V. Kokoropoulos P. Arkadopoulos N.. (2024). Dysbiosis signature of fecal microbiota in patients with pancreatic adenocarcinoma and pancreatic Intraductal papillary mucinous neoplasms. Biomedicines 12. doi: 10.3390/biomedicines12051040, PMID: 38791002 PMC11117863

[ref32] Siegel R. L. Miller K. D. Wagle N. S. Jemal A. (2023). Cancer statistics, 2023. CA Cancer J. Clin. 73, 17–48. doi: 10.3322/caac.21763, PMID: 36633525

[ref33] Song S. J. Lauber C. Costello E. K. Lozupone C. A. Humphrey G. Berg-Lyons D.. (2013). Cohabiting family members share microbiota with one another and with their dogs. eLife 2:e00458. doi: 10.7554/eLife.00458, PMID: 23599893 PMC3628085

[ref34] Torres P. J. Fletcher E. M. Gibbons S. M. Bouvet M. Doran K. S. Kelley S. T. (2015). Characterization of the salivary microbiome in patients with pancreatic cancer. PeerJ 3:e1373. doi: 10.7717/peerj.1373, PMID: 26587342 PMC4647550

[ref35] Uriarte-Navarrete I. Hernandez-Lemus E. De Anda-Jauregui G. (2021). Gene-microbiome Co-expression networks in Colon Cancer. Front. Genet. 12:617505. doi: 10.3389/fgene.2021.617505, PMID: 33659025 PMC7917223

[ref36] Vogtmann E. Han Y. Caporaso J. G. Bokulich N. Mohamadkhani A. Moayyedkazemi A.. (2020). Oral microbial community composition is associated with pancreatic cancer: a case-control study in Iran. Cancer Med. 9, 797–806. doi: 10.1002/cam4.2660, PMID: 31750624 PMC6970053

[ref37] Walton K. Wang T. W. Prutzman Y. Jamal A. Babb S. D. (2020). Characteristics and correlates of recent successful cessation among adult cigarette smokers, United States, 2018. Prev. Chronic Dis. 17:E154. doi: 10.5888/pcd17.200173, PMID: 33301394 PMC7769075

[ref38] Wei A. L. Li M. Li G. Q. Wang X. Hu W. M. Li Z. L.. (2020). Oral microbiome and pancreatic cancer. World J. Gastroenterol. 26, 7679–7692. doi: 10.3748/wjg.v26.i48.7679, PMID: 33505144 PMC7789059

[ref39] Woerner J. Huang Y. Hutter S. Gurnari C. Sanchez J. M. H. Wang J.. (2022). Circulating microbial content in myeloid malignancy patients is associated with disease subtypes and patient outcomes. Nat. Commun. 13:1038. doi: 10.1038/s41467-022-28678-x, PMID: 35210415 PMC8873459

[ref40] Xu Z. Jiang W. Huang W. Lin Y. Chan F. K. L. Ng S. C. (2022). Gut microbiota in patients with obesity and metabolic disorders—a systematic review. Genes Nutr. 17:2. doi: 10.1186/s12263-021-00703-6, PMID: 35093025 PMC8903526

[ref41] Yang J. Ma Y. Tan Q. Zhou B. Yu D. Jin M.. (2023). Gut Streptococcus is a microbial marker for the occurrence and liver metastasis of pancreatic cancer. Front. Microbiol. 14:1184869. doi: 10.3389/fmicb.2023.1184869, PMID: 37389332 PMC10306441

[ref42] Yu Q. Jobin C. Thomas R. M. (2021). Implications of the microbiome in the development and treatment of pancreatic cancer: thinking outside of the box by looking inside the gut. Neoplasia 23, 246–256. doi: 10.1016/j.neo.2020.12.008, PMID: 33418277 PMC7804346

[ref43] Zambirinis C. P. Pushalkar S. Saxena D. Miller G. (2014). Pancreatic cancer, inflammation, and microbiome. Cancer J. 20, 195–202. doi: 10.1097/PPO.0000000000000045, PMID: 24855007 PMC4112373

[ref44] Zhou J. Wang L. Yuan R. Yu X. Chen Z. Yang F.. (2020). Signatures of mucosal microbiome in oral squamous cell carcinoma identified using a random forest model. Cancer Manag. Res. 12, 5353–5363. doi: 10.2147/CMAR.S251021, PMID: 32753953 PMC7342497

[ref45] Zhou W. Zhang D. Li Z. Jiang H. Li J. Ren R.. (2021). The fecal microbiota of patients with pancreatic ductal adenocarcinoma and autoimmune pancreatitis characterized by metagenomic sequencing. J. Transl. Med. 19:215. doi: 10.1186/s12967-021-02882-7, PMID: 34006295 PMC8130326

