# Supplementary figures and images for: Distinct microbiome composition and reduced interactions in patients with pancreatic cancer
Source: Front Microbiol. 2025 Jun 20;16:1555479. doi: 10.3389/fmicb.2025.1555479 (PMC12231506; doi:10.3389/fmicb.2025.1555479)

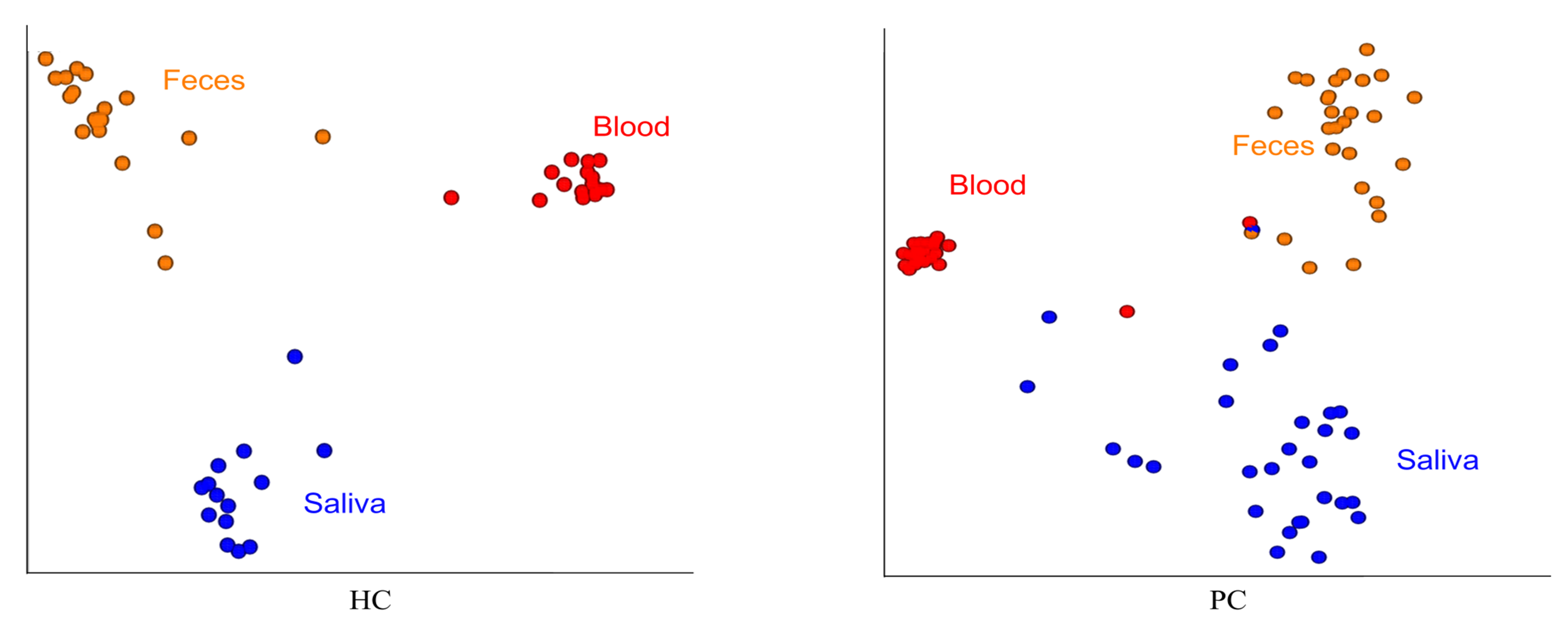

Supplement: SUPPLEMENTARY FIGURE 1 — The microbial composition relationships between the sample types were assessed between healthy controls and patients with pancreatic cancer. Principal coordinate analysis (PCoA) was employed to visualize the relationships between samples using unweighted UniFrac distance matrices. HC, healthy control; PC, patient with pancreatic cancer. Differences in microbial composition between patients with pancreatic cancer and healthy controls. [file Image_1.TIF]
